# Extracellular Matrix Tissue Patch for Septal Defect Repair in Pediatric Cardiac Surgery: A Single-Center Experience

**DOI:** 10.3390/jcm15145744

**Published:** 2026-07-22

**Authors:** Marcin Gładki, Paweł R. Bednarek, Anita Węclewska, Tomasz Urbanowicz, Anna Olasińska-Wiśniewska, Bartłomiej Kociński, Jowita Rosada-Kurasińska, Marek Jemielity

**Affiliations:** 1 Department of Pediatric Cardiac Surgery, Poznan University of Medical Sciences, 60-572 Poznan, Poland; 2 Department of Cardiac Surgery and Transplantology, Poznan University of Medical Sciences, 61-848 Poznan, Poland; 3 Department of Pediatric Anesthesiology and Intensive Care, Poznan University of Medical Sciences, 60-572 Poznan, Poland

**Keywords:** pediatric cardiac surgery, congenital heart defect, atrial septal defect, ventricular septal defect, extracellular matrix

## Abstract

**Background:** Decellularized extracellular matrix (ECM) patches have emerged as a potential alternative to synthetic and autologous materials in pediatric cardiac surgery; however, clinical data on their use in septal defect repair remain limited. **Methods:** This single-center retrospective study evaluated the applicability and early outcomes of ECM scaffolds for pediatric septal defect repair using data from the national cardiac surgery registry. Early postoperative outcomes and perioperative variables were analyzed. **Results:** The study included 72 procedures performed in 68 patients (35 males and 33 females), aged 10 days to 16 years (median age: 187 days; IQR: 105–327 days). Reoperations accounted for 6% of cases. Postoperative complications occurred in 1.4% of patients, and continuous renal replacement therapy was required in 6 (8.8%) patients. Overall mortality was 2.9% (2/68 patients). No statistically significant differences were observed between atrial and ventricular septal defect groups. **Conclusions:** ECM patches appeared to be a safe and effective option for septal defect repair in pediatric cardiac surgery, demonstrating low complication rates and satisfactory early outcomes across different types of congenital heart defects.

## 1. Introduction

Heart septal defects are the most common among congenital heart defects (CHDs). Septal defects comprise a heterogeneous group of congenital heart defects ranging from isolated atrial (ASDs) or ventricular septal defects (VSDs) to complex atrioventricular canal (AVC) malformations associated with additional intracardiac anomalies. VSD is the most prevalent congenital cardiac anomaly in children [1] and can occur both as a single defect or coexists with others to form complex defect syndromes, such as tetralogy of Fallot (TOF). VSD can be located in different places within the septum. The most common defects are located in the membranous part of the ventricular septum. Another group consists of patients in whom VSD is only part of a larger circulatory problem. This group is very diverse, ranging from children diagnosed with TOF to ventricular hypoplasia. VSD in TOF has a characteristic structure—it is called anterior malalignment defect and located under the aorta, resulting from the upward and forward displacement of the conal septum, which causes misalignment of the septal fragments and the formation of a defect in this area. There is also a posterior malalignment defect in patients with interrupted aortic arch (IAA).

Another type of heart defect is ASD. The most common type is an ostium secundum atrial septal defect (ASD II). Depending on the size and shape of the defect, as well as the presence of other cardiovascular defects, a decision is made to close it either interventionally or surgically. Patients with a corrected defect have a very good prognosis and return to fully normal functioning [2].

The clinical situation differs in children with AVC defects, which also involves the atrioventricular valves. According to Wakai [3], there are three types of this defect: (1) the first group consists of partial AVC defects, the most common manifestation of which is an ostium primum atrial septal defect (ASD I) with a cleft in the anterior leaflet of the mitral valve, (2) the second group is complete AVC defect, characterized by the presence of a common atrioventricular valve and ASD that smoothly transitions into an inflow tract VSD, (3) the third group consists of children with transitional AVC, which is an intermediate form between partial and complete. Most AVC (both partial, transitional, and complete) are amenable to surgical correction. In very young children with symptomatic circulatory failure, a two-stage treatment is used: the first-stage procedure consists of pulmonary artery banding (PAB), followed by defect correction surgery a few months later. A similar approach is also used in some patients with isolated VSD and severe symptomatic circulatory failure. Each subsequent intervention in the chest increases the risk of complications. Among children with heart septal defects (without other cardiovascular anomalies), those with complete AVC defect constitute the group of the most severely affected patients. Symptoms of circulatory failure in children with partial AVC defect appear much later, and surgical correction is usually performed in a single stage. Approximately 70–80% of children with atrioventricular canal defects are reported to have trisomy 21 (Down syndrome) [4,5]. These children have a characteristic anatomical configuration of the defect, which allows for a significantly better hemodynamic effect after surgery. However, Down syndrome is associated with the coexistence of many health problems outside the circulatory system, which reduces the quality and length of life of these patients.

The strategy for the treatment of CHDs is predicated on surgical intervention at an early stage in life [6]. A substantial proportion of these cases necessitate the implementation of reconstructive techniques that involve the utilisation of prosthetic materials. Despite the favourable perioperative survival rate, the incidence of reoperation due to graft failure remains considerable. In order to achieve complete therapeutic success, it is necessary to utilize a material that maximises the chances of reconstructing the tissue of the heart, pericardium or vessels. Therefore, it is imperative that the treatment should emulate the natural physiological properties of the repaired tissues to the greatest extent possible. The ideal cardiovascular patch should combine excellent handling characteristics with biocompatibility, facilitate the healing process, exhibit an anti-inflammatory effect, and be resistant to calcification and infection. Additionally, it should possess plasticity and flexibility, as well as a high degree of resistance to stress, thereby enabling the surgeon to model a desired shape. The aforementioned properties appear to be challenging to accomplish. The commonly used prosthetic materials in pediatric cardiac surgery, including synthetic (polyethylene terephthalate (PET), e.g., Dacron^®^ (Chester, VA, USA), or polytetrafluoroethylene (PTFE), e.g., GoreTex^®^, (Newark, DE, USA)) and biological (e.g., autologous pericardium, allografts, xenografts) materials, do not meet the listed requirements of an ideal cardiovascular substitute, particularly with regard to growth potential, resistance to calcification, and long-term remodeling capacity [7].

Recently, however, bioscaffolds have been created from decellularized extracellular matrix (ECM) due to developments in tissue engineering. The aim was to create a transitional scaffold for host cells to colonize, allowing the prosthetic structures to regenerate naturally (a process called “constructive remodeling”) and grow as the child’s body develops. Commercially available ECM is obtained from a variety of tissues [8]. In congenital cardiac surgery, bioscaffolds obtained from the small intestine submucosa (SIS) have been particularly widely used. The occurrence of structural remodeling has been demonstrated by both in vivo studies in animal models [9,10] and clinical studies in the pediatric population [11,12,13]. The concept of whole-organ decellularization and biologic scaffold remodeling has been extensively investigated in regenerative medicine and tissue engineering [14]. However, literature data on the use of SIS-ECM in congenital cardiac surgery is still limited. In the following paper, we describe our experience in the use of ProxiCor^®^ SIS-ECM (Elutia, Gaithersburg, MD, USA) in diverse types of intracardiac repairs.

Heart septal defects constitute a very diverse group, and the choice of material for their closure is a topic of discussion among cardiac surgeons. Consequently, reconstructive procedures vary substantially in anatomical complexity, operative time, and the amount of patch material required. These differences provide an opportunity to evaluate the clinical performance of implantable materials under a broad spectrum of surgical conditions. Although ECM scaffolds have been increasingly adopted in congenital cardiac surgery for repair of vessel defects, published clinical experience with ProxiCor^®^ for septal defect repair remains limited and is largely restricted to small observational studies. Consequently, evidence regarding its early clinical performance across different anatomical locations and varying complexity of congenital heart defects remains scarce. In this article, we present the results of using ProxiCor^®^ patches to close heart septal defects in a pediatric population.

Among the potential applications of patches in surgical procedures of pediatric cardiac surgery is the correction of relatively simple CHD, such as closure of ASD and VSD. In cases of primary correction, implantation of an autologous patch derived from the patient’s own pericardium appears to be the optimal choice. Conversely, this approach may not be suitable for reoperation of patients with complex heart defects who have previously undergone staged treatment. In cases where postoperative adhesions in the mediastinum have formed, it can be challenging to obtain a pericardial patch of sufficient quality and mechanical strength. The use of implantable materials, particularly in pediatric cardiac surgery, necessitates the highest possible integrity of the implant in conjunction with native tissue. A range of ECM products are currently available on the market, including a standard three-layer ProxiCor^®^ patch and a thin-walled two-layer version (CorMatrix^®^ patch (CorMatrix Cardiovascular, Inc., Roswell, GA, USA)).

## 2. Materials and Methods

The goal of this single-center retrospective analysis was to assess the applicability and early outcomes of decellularized ECM scaffolds for heart septal defect repair in children using data from the national cardiac surgery registry (pol. Krajowy Rejestr Operacji Kardiochirurgicznych—KROK, Children’s Memorial Health Institute, Warsaw, Poland; https://krok.csioz.gov.pl/krok/, accessed on 11 September 2025). The objective of the present study does not compare ProxiCor^®^ with other patch materials but evaluates its feasibility, safety, and early clinical performance in routine pediatric congenital cardiac surgery. It additionally assesses whether early outcomes differ according to the anatomical site of implantation (interatrial vs. interventricular septum) and the complexity of the underlying congenital heart defect.

The first use of the ECM in the Department of Pediatric Cardiac Surgery, Poznan University of Medical Sciences (Poznań, Greater Poland Voivodeship, Poland) was reported in 2023. This study presents the single-center results of the application of ECM-based implantable materials in patients with heart septal defects.

The study included 68 patients who underwent a total of 72 surgical procedures, as some patients required more than one operation during the study period. All 72 (100%) surgical procedures were performed from a standard access via median sternotomy approach using extracorporeal circulation (ECC). The arterial cannula was inserted in the ascending aorta (Ao). Venous cannulation was performed using two cannulas as follows: the first, the straight cannula, was inserted through the right atrium (RA) into the superior vena cava (SVC); and the second, the curved Pacifico cannula, was inserted directly into the inferior vena cava (IVC). The heart was stopped with antegrade infusion of cold blood del Nido cardioplegic solution into the aortic bulb.

### 2.1. Ethical Considerations

The Bioethics Committee at Poznan University of Medical Sciences (Poland) Statement No. KB–576/25 (10 September 2025) confirms that according to Polish law and Good Clinical Practice (GCP) regulations, this research does not require the approval of the bioethics committee. The consent of individual patients was waived due to the non-experimental nature of the study based on retrospective analysis of medical records, as determined by the institutional bioethics committee board. All data processing was conducted in compliance with the European Union (EU) General Data Protection Regulation (GDPR) and Polish national law. Anonymity and confidentiality were ensured.

### 2.2. Study Population

The study included the population of 68 consecutive patients admitted to the Department of Pediatric Cardiac Surgery, Poznan University of Medical Sciences (Poznań, Greater Poland Voivodeship, Poland) who underwent 72 surgical procedures for heart septal defect repair between 2023 and 2024, following the introduction of ProxiCor^®^ for Cardiac Tissue Repair (CTR) (Elutia, Inc., Gaithersburg, MA, USA; formerly Aziyo Biologics, Inc., Roswell, NM, USA) into the portfolio of available implantable patches. Patients were consecutively enrolled based on the indication for septal defect closure using a ProxiCor^®^ patch. Exclusion criteria included incomplete medical records, the use of an autologous or synthetic patch for defect repair, or defect closure performed with a direct suture technique not involving patch implantation.

Two main groups were defined according to the anatomical localization of the septal defect (i.e., the site of patch implantation): (1) interatrial septal (IAS) defect and (2) interventricular septal (IVS) defect. Each group was subsequently subdivided according to the hemodynamic nature of the CHD into two subgroups: (1) isolated simple defects and (2) defects associated with complex CHD.

Postoperative complications were predefined and analyzed separately as ECM-related events directly attributable to the patch (e.g., residual shunt or patch dysfunction) and as general surgery-related complications not specific to the patch, including acute kidney injury (AKI), need for continuous renal replacement therapy (CRRT), arrhythmias, infection, and other postoperative adverse events.

### 2.3. Materials

The ProxiCor^®^ Cardiac Tissue Repair (CTR) patch (Elutia Inc., Gaithersburg, MD, USA) is a decellularized ECM scaffold derived from porcine small intestinal submucosa (SIS). The proprietary manufacturing process removes cellular components while preserving the native three-dimensional ECM architecture composed predominantly of collagen, elastin, glycosaminoglycans, and naturally occurring bioactive molecules. The material is supplied as a sterile, non-crosslinked, ready-to-use three-layer patch intended to provide immediate mechanical support while serving as a temporary scaffold for host cell infiltration, neovascularization, and constructive tissue remodeling. Unlike permanent synthetic materials, the scaffold is designed to undergo gradual integration with the patient’s own tissue. In our institution, ProxiCor^®^ has become the preferred patch material for elective septal defect reconstruction because of its favorable handling characteristics, flexibility, ease of suturing, and potential for biological integration.

### 2.4. Statistical Analysis

Data distribution was assessed using the Shapiro–Wilk test. Continuous variables were summarized as median with interquartile range (IQR; Q1–Q3) and range (minimum–maximum) for non-normally distributed data, and as mean with standard deviation (SD) for approximately normally distributed data. Categorical parameters are reported as absolute counts and percentages. Statistical analysis was conducted with the Jeffreys’s Amazing Statistics Program (JASP) statistical software (version 0.13.1, JASP Team, Amsterdam, The Netherlands, 2020).

Between-group comparisons for continuous variables were performed using the Mann–Whitney U test for non-normally distributed data and Student’s *t*-test for normally distributed data, as appropriate. Categorical variables were compared using the χ^2^ test or Fisher’s exact test, as appropriate. A two-sided *p*-value < 0.05 was considered statistically significant.

## 3. Results

The study population consisted of 68 patients, including 33 (49%) males and 35 (51%) females, aged 10 days to 16 years (median age: 187 days; IQR: 105–327 days). Among them, 3 (4%) were newborns (≤28 days) with a median age of 18 (IQR: 12–24) days, 50 (74%) were infants aged 1–12 months with a median age of 5.0 (IQR: 3.0–7.5) months, and 15 (22%) were children older than 12 months with a median age of 24 (IQR: 18–36) months. The median body weight and height at the time of surgery were 6.2 (IQR: 4.8–8.4) kg and 63 (IQR: 55–74) cm, respectively (Table 1).

In the IAS defect group of 15 patients with ASD (operated on using a technique other than direct continuous suture technique closure of the defect with a continuous suture or implantation of an autologous patch closing the defect), the ProxiCor^®^ patch was implanted 17 times: in 5 patients with isolated ASD II, in 1 patient with sinus venosus atrial septal defect (ASD sv) associated with partial anomalous pulmonary venous return (PAPVR), in 2 patients with total anomalous pulmonary venous return (TAPVR), in 5 patients with isolated ASD I partial AVC, 3 times in 1 patient with ASD I partial AVC associated with hypoplastic aortic arch (HAA), and in 1 patient with ASD associated with TOF. The total number of 17 patch implantations exceeded the number of 15 patients because 1 patient with AVC associated with HAA underwent 3 surgical procedures: the primary operation of correction of partial AVC followed by 2 reoperations on mitral valve performed via a transseptal approach, with subsequent reimplantation of patch for ASD closure each time.

In the IVS defect group of 53 patients with VSD, the ProxiCor^®^ patch was implanted 55 times: 30 times in 29 patients with isolated VSD, in 19 patients for VSD closure in complete correction of TOF, 3 times for VSD closure in 2 patients with transposition of the great arteries (TGA), and in 3 patients for closure of VSD associated with complex aortic arch defect repair. The total number of 55 patch implantations exceeded the number of 53 patients because 1 patient with isolated VSD underwent 2 surgical procedures: the primary operation for VSD closure followed by 1 reoperation for residual VSD closure, and 1 patient with VSD associated with Taussig–Bing anomaly underwent 2 surgical procedures: the primary operation of correction of Taussig–Bing anomaly with VSD closure followed by 1 reoperation, with subsequent reimplantation of patch for VSD closure each time. Table 2 summarizes distribution of patients and surgeries according to primary diagnosis.

A total of 72 surgical procedures were performed, including 17 patch implantations in the IAS defect group and 55 in the IVS defect group. Reoperations accounted for 4 (6%) procedures. 

The median cardiopulmonary bypass (CPB) time was 97 (IQR: 67–136) minutes, and the median aortic cross-clamp (AoX) time was 53 (IQR: 33–69.5) minutes. All 72 (100%) procedures were performed using standard extracorporeal circulation with cold blood del Nido cardioplegia. Detailed perioperative and hospitalization data are presented in Table 3.

**Figure 1:**
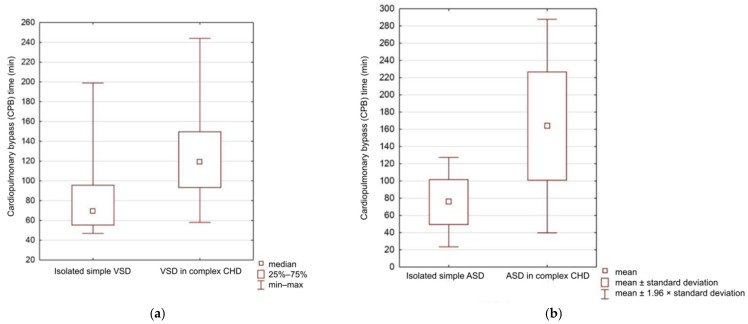
CPB time: (**a**) IVS group. (**b**) IAS group. CPB—cardiopulmonary bypass, VSD—ventricular septal defect, ASD—atrial septal defect, CHD—congenital heart defect.

**Figure 2:**
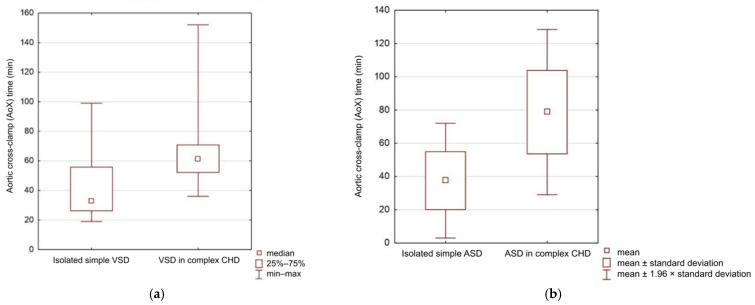
AoX time: (**a**) IVS groups. (**b**) IAS group. AoX—aortic cross-clamp, VSD—ventricular septal defect, ASD—atrial septal defect, CHD—congenital heart defect.

Procedures involving complex CHD (including TOF, TGA, and aortic arch anomalies) were associated with longer CPB (Figure 1) and AoX (Figure 2) times compared with isolated defects, reflecting higher surgical complexity.

### Early Postoperative Outcomes in Both Groups

Median duration of mechanical ventilation time was 23.5 (IQR: 7–112.5) h, median duration of postoperative intensive care unit (ICU) stay was 82 (IQR: 35–237.5) h, and median total hospitalization time was 10 (IQR: 7–16.5) days in both groups.

Prolonged (>72 h) mechanical ventilation support was required in 19 (28%) patients, predominantly in those with complex CHD. Postoperative AKI requiring CRRT using continuous veno-venous hemodiafiltration (CVVHDF) protocol occurred in 6 (8.8%) patients.

There were no cases of early patch-related structural failure, rupture, or significant dehiscence. Residual shunts requiring reintervention were observed in 2 (3%) cases, both in the IVS group. Reoperations were performed in 4 (6%) of cases. Postoperative complications occurred in 1 patient (1.4%). Overall mortality was 2.9% (2 patients), including 1 patient in the IVS group (1.8%) and 1 patient in the IAS group (5.9%).

No statistically significant differences were observed between the IAS and IVS groups with regard to demographic parameters, perioperative variables, or early postoperative outcomes (Table 4). A trend toward longer CPB and aortic cross-clamp times in the IAS group was observed but did not reach a threshold of statistical significance (*p* = 0.089 and *p* = 0.072, respectively).

In subgroup analysis within the IVS cohort, patients with complex CHD had significantly longer CPB and aortic cross-clamp times, as well as longer mechanical ventilation, ICU stay, and hospitalization (all *p* < 0.05).

Similarly, in the IAS group, patients with complex defects demonstrated significantly longer operative times and postoperative recovery parameters compared to those with isolated defects (*p* < 0.05).

As the compared variables in both groups did not follow a normal distribution, non-parametric statistical methods were applied.

When comparing the IAS and IVS groups, a statistically significant association was observed for CVVHDF use, whereas no significant association was found with mortality risk (Table 4).

In the IAS group, all results were statistically significant due to the heterogeneity of the ASD patient group, particularly with regard to the wide age range (Table 5).

In the IVS group, despite longer operative times, there were no statistically significant differences in the number of complications or mortality (Table 6).

## 4. Discussion

The primary objective of this study does not demonstrate superiority of ProxiCor^®^ over alternative patch materials but evaluates its early clinical applicability in routine pediatric congenital cardiac surgery. Because ProxiCor^®^ became the preferred implantable patch in our institution following its introduction, a contemporary comparison with previously used materials was not feasible. Instead, we assessed its performance across different anatomical implantation sites and varying levels of surgical complexity encountered in everyday clinical practice. Our experience demonstrated that the use of ECM patches fulfills its purpose and performs well in two aspects: both the anatomical location (atrial vs. ventricular) and the complexity nature of the underlying CHD.

In our study, we demonstrated that the use of decellularized ECM patches for septal defect repair in pediatric patients was associated with favorable early outcomes and a low incidence of complications.

We found no significant differences between IAS and IVS groups in terms of perioperative outcomes, suggesting that ECM patches can be effectively used regardless of defect location. Although there was a trend toward longer CPB and AoX times in the IAS group, this finding most likely reflects the increased anatomical and technical complexity of defects included in this subgroup rather than any intrinsic limitation of the implanted material itself. In particular, patients with atrioventricular canal defects, anomalous pulmonary venous return, and associated aortic arch abnormalities frequently require more extensive intracardiac reconstruction and prolonged myocardial ischemic time. Previous studies demonstrated that operative complexity in congenital heart surgery is strongly associated with prolonged CPB and aortic cross-clamp duration, which subsequently translates into longer postoperative ventilation, ICU stay, and overall recovery time [15]. Importantly, despite longer operative times in more complex repairs, no increase in ECM-related complications was observed in our cohort, supporting the feasibility and safety of ECM patch implantation even in technically demanding congenital cardiac procedures.

Subgroup analysis demonstrated that patients with complex CHD required significantly longer operative times and postoperative support. This finding is expected and consistent with the well-established relationship between CHD complexity and postoperative morbidity in pediatric cardiac surgery [16,17]. Previous studies demonstrated that surgical complexity in congenital heart surgery is strongly associated with prolonged cardiopulmonary bypass duration, extended mechanical ventilation, longer ICU stay, and increased postoperative morbidity. Importantly, the use of ECM patches did not appear to negatively influence these outcomes.

The observed low rate of postoperative complications (1.4%) and CRRT in cardiac surgery-associated AKI (8.8%) further support the safety profile of ECM patches in this setting, considering the high proportion of patients with complex CHD and reoperations, which appears comparable to previously reported high-risk pediatric cardiac surgery cohorts. Previous studies reported that severe AKI requiring CRRT occurs in approximately 4–10% of high-risk pediatric cardiac surgical populations and is associated with substantial morbidity [18]. Increased surgical complexity, prolonged cardiopulmonary bypass exposure, and postoperative low cardiac output syndrome are major determinants of respiratory and renal dysfunction following pediatric cardiac surgery. AKI requiring CRRT remains one of the most severe postoperative complications in children undergoing repair of complex CHD and has been associated with prolonged ICU stay and increased morbidity [18]. The observed association between prolonged ventilation, AKI, and complex CHD supports the validity of our cohort as representative of contemporary high-risk pediatric cardiac surgery practice.

The overall mortality rate of 2.9% observed in our cohort is comparable to previously reported outcomes in pediatric cardiac surgery and reflects the inclusion of patients with complex CHD [11,12]. Padalino et al. demonstrated favorable early and mid-term outcomes following ECM scaffold implantation in congenital cardiac and vascular reconstructive surgery, with low rates of ECM-related reintervention and satisfactory tissue integration [13]. Importantly, no patch-related structural complications were observed, supporting the safety of ECM materials in the early postoperative period.

These findings are consistent with our own institutional experience in previous studies focused on aortic arch and pulmonary artery reconstruction using the same material [19,20].

From a clinical perspective, ECM-based materials offer several theoretical advantages over synthetic patches, including improved biocompatibility and the potential for constructive remodeling. These features may be particularly relevant in pediatric patients, where growth and tissue integration are critical. Nevertheless, the present results should not be interpreted as evidence of superior long-term biological performance of ProxiCor^®^. Characteristics such as tissue remodeling, calcification resistance, and growth potential require dedicated prospective studies with mid- and long-term follow-up before definitive conclusions can be drawn.

### Strengths and Limitations

The main strength of this study is the inclusion of a consecutive cohort reflecting real-world clinical practice. However, limitations include the retrospective design, the single-center nature of the study, which reflects the experience of a tertiary referral pediatric cardiac surgery center and was dictated by the lack of access to detailed patient-level data from other national institutions, and relatively short follow-up restricted the analysis to early outcomes. The cohort was clinically heterogeneous, including both simple and complex congenital heart defects, which limits attribution of outcomes solely to the ECM patch and may have been influenced by anatomical complexity, cardiopulmonary bypass duration, and baseline surgical risk. Furthermore, no contemporary control group was available because ProxiCor^®^ became the preferred patch material for elective septal defect reconstruction at our institution, largely replacing previously used synthetic and biological alternatives. Consequently, the absence of a control group precludes feasible direct comparison with other patch materials. A systematic review by Mosala Nezhad et al. highlighted the promising regenerative potential of SIS-ECM materials while emphasizing the limited availability of long-term clinical data [21].

Several subgroup comparisons were limited by small sample sizes and low statistical power; absence of statistically significant differences should not be interpreted as evidence of equivalence.

## 5. Conclusions

Decellularized ECM patches may represent a feasible and apparently safe option for septal defect repair in pediatric cardiac surgery, demonstrating favorable early postoperative outcomes observed in this single-center retrospective cohort across both simple and complex congenital heart defects. The biological characteristics of ProxiCor^®^ support its use as a regenerative scaffold while providing adequate mechanical support during cardiac tissue healing. Their use is associated with low complication rates and satisfactory early outcomes, regardless of defect type.

However, the present study evaluates only early postoperative outcomes. The clinical heterogeneity of the study population and the lack of a comparator group limit definitive conclusions regarding comparative effectiveness. Nevertheless, confirmation of these promising findings requires further prospective multicenter studies with longer follow-up echocardiographic and clinical surveillance investigations directly comparing other different currently available patch materials to determine whether the theoretical biological advantages of ECM scaffolds translate into superior tissue remodeling, durability, resistance to calcification, growth potential, and long-term performance.

## Figures and Tables

**Table 1:** Demographic and clinical characteristics.

Demography	Parameters	Group (*n* = 68)
Sex	Males (*n*) (%)/females (*n*) (%)	33 (49)/35 (51)
Age	1. Newborns (≤28 days of age) (*n*) (%)	3 (4)
	median age (days) (IQR)	18 (12–24)
	2. Infants (1–12 months of age) (*n*) (%)	50 (74)
	median age (months) (IQR)	5.0 (3.0–7.5)
	3. Children (>12 months of age) (*n*) (%)	15 (22)
	median age (months) (IQR)	24 (18–36)
Anthropometry	Median weight (kg) (IQR)	6.2 (4.8–8.4)
	Median height (cm) (IQR)	63 (55–74)

**Table 2:** Study groups according to primary diagnosis and number of patients and surgeries.

Group	Subgroup (*n*)	Primary Diagnosis	Patients (*n*)	Surgeries (*n*)
IAS	Isolated simple ASD (5)	ASD II	5	5
	ASD associated with complex CHD (10)	ASD + PAPVR	1	1
		ASD + TAPVR	2	2
		ASD I	5	5
		ASD + TOF	1	1
		ASD + HAA	1	3
IVS	Isolated simple VSD (29)	VSD	29	30
	VSD associated with complex CHD (24)	VSD + TOF	19	19
		VSD + D-TGA	1	1
		VSD + Taussig-Bing	1	2
		VSD + HAA	3	3

IAS—interatrial septum, IVS—interventricular septum, CHD—congenital heart defect, ASD—atrial septal defect, PAPVR—partial anomalous pulmonary venous return, TAPVR—total anomalous pulmonary venous return, TOF—tetralogy of Fallot, HAA—hypoplastic aortic arch, VSD—ventricular septal defect, D-TGA—dextro-transposition of the great arteries.

**Table 3:** Intraoperative parameters and early postoperative outcomes.

Parameters		Group (*n* = 68)
Surgery time	Overall (minutes) (median) (IQR)	240 (175–277)
	CPB (minutes) (median) (IQR)	97 (67–136)
	Aortic cross-clamp (minutes) (median) (IQR)	53 (33–69.5)
Survival	Overall (*n*) (%)	66 (97.1)
Hospitalization	Mechanical ventilation (hours) (median) (IQR)	23.5 (7–112.5)
	Prolonged mechanical ventilation * (*n*) (%)	19 (28)
	ICU LOS (hours) (median) (IQR)	82 (35–237.5)
	Overall LOS (days) (median) (IQR)	10 (7–16)

CPB—cardiopulmonary bypass, ICU—intensive care unit, LOS—length of stay. * >72 h.

**Table 4:** IAS group and IVS group data.

Variable	IAS Group (*n* = 15)	IVS Group (*n* = 53)	*p*
Sex (male/female) (*n*) (%)	7 (47)/8 (53)	26 (49)/27 (51)	0.88352
Age (days) (min–max)	10–5854 (median: 278, IQR: 122–445)	11–4527 (median: 181, IQR: 104–251)	0.329738 ^a^
Weight (kg) (min–max)	3.3–49 (median: 7, IQR: 4.5–9.3)	3.1–28 (median: 6.2, IQR: 5.2–7.8)	0.559477 ^a^
Height (cm) (min–max)	54–160 (median: 68, IQR: 55–80)	51–138 (median: 68, IQR: 62–73)	0.800613 ^a^
CPB time (min) (min–max)	47–293 (median: 122, IQR: 71–171)	47–244 (median: 92, IQR: 63–129)	0.089613 ^a^
AoX time (min) (min–max)	22–125 (median: 69, IQR: 38–83)	19–152 (median: 50, IQR: 32–64)	0.072285 ^a^
Mechanical ventilation (hours) (min–max)	2–387 (median: 25, IQR: 7–125)	2–900 (median: 23, IQR: 7–105)	0.760220 ^a^
ICU LOS (hours) (min–max)	11–438 (median: 155, IQR: 57–346)	31–894 (median: 81, IQR: 35–225)	0.433040 ^a^
Hospital LOS (days) (min–max)	4–59 (median: 9, IQR: 7–21)	6–41 (median: 10, IQR: 7–16)	0.544017 ^a^
Postoperative AKI (*n*) (%)	4 (24)	2 (4)	0.02488
Postoperative complications (*n*) (%)	0 (0)	1 (1.8)	1.0
Survival (*n*) (%)	14 (93)	52 (98)	0.41901

IAS—interatrial septum, IVS—interventricular septum, ICU—intensive care unit, LOS—length of stay, CPB—cardiopulmonary bypass, AoX—aortic cross-clamp, AKI—acute kidney injury. ^a^ Mann–Whitney U test (with continuity correction).

**Table 5:** IAS group.

Variable	Isolated Simple ASD (*n* = 5)	ASD in Complex CHD (*n* = 12)	*p*
Age (days) (min–max)	204–5854 (median: 706.5, IQR: 408–1056)	10–522 (median: 127, IQR: 21–323)	0.010382 ^a^
Weight (kg) (min–max)	7–49 (median: 10.9, IQR: 7.9–13.5)	3.3–9.3 (median: 5.2, IQR: 3.8–7.2)	0.004087 ^a^
Height (cm) (min–max)	65–160 (median: 88.5, IQR: 80–95)	54–76 (median: 64, IQR: 55–68)	0.005392 ^a^
CPB time (min) (min–max)	47–116 (mean: 75.5 ± 27)	62–293 (mean: 164 ± 63)	0.005630 ^b^
AoX time (min) (min–max)	22–69 (mean: 37.5 ± 18)	38–125 (mean: 79 ± 25)	0.003073 ^b^
Mechanical ventilation time (hours) (min–max)	2–24 (median: 5, IQR: 4–7)	12–387 (median: 100, IQR: 25–215)	0.002161 ^a^
ICU LOS (hours) (min–max)	11–297 (median: 33, IQR: 31–155)	57–438 (median: 224, IQR: 82–416)	0.023739 ^a^
Hospital LOS (days) (min–max)	4–14 (median: 6.5, IQR: 5–9)	7–59 (median: 17, IQR: 9–48)	0.009872 ^a^

ASD—atrial septal defect, CHD—congenital heart defect, ICU—intensive care unit, LOS—length of stay, CPB—cardiopulmonary bypass, AoX—aortic cross-clamp. ^a^ Mann–Whitney U test (with continuity correction), ^b^ Student’s t-test (for independent groups).

**Table 6:** IVS group.

Variable	Isolated Simple VSD (*n* = 30)	VSD in Complex CHD (*n* = 25)	*p*
Age (days) (min–max)	58–3856 (median: 155, IQR: 104–241)	11–4527 (median: 204, IQR: 106–251)	0.531655 ^a^
Weight (kg) (min–max)	3.9–26.5 (median: 6, IQR: 5–8)	3.1–28 (median: 6.5, IQR: 5–7)	0.799754 ^a^
Height (cm) (min–max)	52–138 (median: 64, IQR: 61–70)	51–138 (median: 68, IQR: 62–73)	0.300785 ^a^
CPB time (min) (min–max)	47–199 (median: 69.5, IQR: 55–96)	58–244 (median: 119, IQR: 93–150)	0.000028 ^a^
AoX time (min) (min–max)	19–99 (median: 33, IQR: 26–56)	36–152 (median: 61, IQR: 52–71)	0.000048 ^a^
Mechanical ventilation time (hours) (min–max)	2–817 (median: 9, IQR: 6–30)	3–900 (median: 33, IQR: 9–193)	0.006785 ^a^
ICU LOS (hours) (min–max)	33–813 (median: 46.5, IQR: 35–131)	31–894 (median: 144, IQR: 58–272)	0.046156 ^a^
Hospital LOS (days) (min–max)	6–34 (median: 7.5, IQR: 7–15)	6–41 (median: 14, IQR: 8–21)	0.048012 ^a^

VSD—ventricular septal defect, ICU—intensive care unit, LOS—length of stay, CPB—cardiopulmonary bypass, AoX—aortic cross-clamp. ^a^ Mann–Whitney U test (with continuity correction).

## Data Availability

The data presented in this study are available on request from the corresponding author.

## Abbreviations

The following abbreviations are used in this manuscript:

ASD	Atrial septal defect
ASD I	Ostium primum atrial septal defect
ASD II	Ostium secundum atrial septal defect
ASD sv	Sinus venosus atrial septal defect
AKI	Acute kidney injury
AoX	Aortic cross-clamp
AVC	Atrioventricular canal
CHD	Congenital heart defect
CPB	Cardiopulmonary bypass
CRRT	Continuous renal replacement therapy
CVVHDF	Continuous veno-venous hemodiafiltration
ECC	Extracorporeal circulation
ECM	Extracellular matrix
HAA	Hypoplastic aortic arch
IAA	Interrupted aortic arch
IAS	Interatrial septum
ICU	Intensive care unit
IVS	Interventricular septum
KROK	Krajowy Rejestr Operacji Kardiochirurgicznych–national cardiac surgery registry
PAB	Pulmonary artery banding
PAPVR	Partial anomalous pulmonary venous return
SIS	Small intestine submucosa
TAPVR	Total anomalous pulmonary venous return
TGA	Transposition of the great arteries
TOF	Tetralogy of Fallot
VSD	Ventricular septal defect
